# Supplementary material for: Nutrient dominance governs the assembly of microbial communities in mixed nutrient environments
Source: eLife. 2021 Apr 20;10:e65948. doi: 10.7554/eLife.65948 (PMC8057819; doi:10.7554/eLife.65948)
Supplement: Supplementary file 1. — (a) Carbon sources used in this study. (b) Taxonomy of strains used in the growth rate assay and community they were isolated from. [file elife-65948-supp1.docx]

**Supplementary file 1a. Carbon sources used in this study**

| **Carbon source** | **Supplier** | **Reference** | **pH (in M9)** | **Final concentration (mM)** |
| --- | --- | --- | --- | --- |
| D-Glucose | VWR | 0188-500 | 6.83 | 11.67 |
| D-Cellobiose | Sigma | 22150-10G | 6.84 | 5.83 |
| D-Fructose | Acros Organics | 161355000 | 6.79 | 11.67 |
| D-Ribose | Acros Organics | AC132361000 | 6.81 | 13.99 |
| Glycerol (80%, w/v) | Teknova | G8797 | 6.81 | 23.33 |
| Sodium Succinate hexahydrate | Alfa Aesar | 419A3 | 6.84 | 17.50 |
| Sodium hydrogen fumarate | Alfa Aesar | B24683 | 6.11 | 17.50 |
| Sodium benzoate | Alfa Aesar | A15946 | 6.80 | 10.0 |
| L-Glutamine 200mM (29.23 mg/mL) | Sigma | G7513-100ML | 6.80 | 14.0 |
| Glycine | Sigma | G7126-100G | 6.82 | 35.0 |

**Supplementary file 1b. Taxonomy of strains used in the growth rate assay and community they were isolated from.**

| **Family** | **Genus** | **Transfer_CarbonSource_Inoculum_Replicate** |
| --- | --- | --- |
| Enterobacteriaceae | Raoultella | T10_glucose_I1_R2 |
| Enterobacteriaceae | Citrobacter | T10_glucose-cellobiose_I1_R1 |
| Enterobacteriaceae | Klebsiella | T10_glucose-cellobiose_I1_R1 |
| Enterobacteriaceae | Citrobacter | T10_succinate_I2_R1 |
| Enterobacteriaceae | Enterobacter | T10_succinate_I2_R1 |
| Enterobacteriaceae | Klebsiella | T10_succinate_I2_R4 |
| Enterobacteriaceae | Raoultella | T10_glutamine_I2_R2 |
| Moraxellaceae | Acinetobacter | T10_succinate_I2_R1 |
| Moraxellaceae | Acinetobacter | T10_succinate_I2_R1 |
| Moraxellaceae | Acinetobacter | T10_succinate_I2_R4 |
| Moraxellaceae | Acinetobacter | T10_succinate_I2_R4 |
| Moraxellaceae | Acinetobacter | T10_glutamine_I2_R2 |
| Moraxellaceae | Acinetobacter | T10_glutamine_I2_R2 |
| Pseudomonadaceae | Pseudomonas | T10_glutamine_I2_R3 |
| Pseudomonadaceae | Pseudomonas | T10_ribose_I1_R1 |
| Pseudomonadaceae | Pseudomonas | T10_benzoate_I1_R3 |
| Pseudomonadaceae | Pseudomonas | T10_fumarate_I2_R2 |
| Pseudomonadaceae | Pseudomonas | T10_benzoate_I2_R3 |
| Rhizobiaceae | Rhizobium | T10_succinate_I2_R1 |
| Rhizobiaceae | Rhizobium | T10_succinate_I2_R1 |
| Rhizobiaceae | Rhizobium | T10_succinate_I2_R4 |
| Rhizobiaceae | Rhizobium | T10_glutamine_I2_R2 |
